# Validation of Planning Target Volume Margins by Analyzing Intrafractional Localization Errors for 14 Prostate Cancer Patients Based on Three-Dimensional Cross-Correlation between the Prostate Images of Planning CT and Intrafraction Cone-Beam CT during Volumetric Modulated Arc Therapy

**DOI:** 10.1155/2014/960928

**Published:** 2014-05-22

**Authors:** Kenshiro Shiraishi, Masahiko Futaguchi, Akihiro Haga, Akira Sakumi, Katsutake Sasaki, Kentaro Yamamoto, Hiroshi Igaki, Kuni Ohtomo, Kiyoshi Yoda, Keiichi Nakagawa

**Affiliations:** ^1^Department of Radiology, University of Tokyo Hospital, 7-3-1 Hongo, Bunkyo-ku, Tokyo 113-8655, Japan; ^2^Elekta KK, Tokyo 108-0023, Japan

## Abstract

Time-averaged intreatment prostate localization errors were calculated, for the first time, by three-dimensional prostate image cross-correlation between planning CT and intrafraction kilovoltage cone-beam CT (CBCT) during volumetric modulated arc therapy (VMAT). The intrafraction CBCT volume was reconstructed by an inhouse software after acquiring cine-mode projection images during VMAT delivery. Subsequently, the margin between a clinical target volume and a planning target volume (PTV) was obtained by applying the van Herk and variant formulas using the calculated localization errors. The resulting PTV margins were approximately 2 mm in lateral direction and 4 mm in craniocaudal and anteroposterior directions, which are consistent with the margin prescription employed in our facility.

## 1. Introduction

It is known that prostate organ moves when rectal volume changes. Direct mechanical forces produced by rectal filling such as gas or stool can explain this phenomenon [1]. Because of this internal prostate organ movement, it is desirable to reposition the patient couch by registering the prostate organ between pretreatment cone-beam CT (CBCT) and planning CT images rather than bone-to-bone registration for reducing treatment margins.

Another aspect is prostate motion during treatment due to possible rectal volume changes. Intrafraction prostate motion analysis was performed by various ways including ultrasound imaging before and after treatment [1], embedded fiducial markers with a portal imager [2–4], or electromagnetic coil system [5]. The reported prostate displacement during treatment exceeded a few millimeters with an increasing probability for a longer delivery time [5], indicating that a planning target volume (PTV) margin would be underestimated if the margin was based on pretreatment positioning errors using CBCT imaging. On the other hand, a recent study revealed that postdelivery CBCT imaging overestimated the localization errors due to the delay between the end of treatment delivery and posttreatment CBCT [6], and the author suggested a use of combined CBCT acquisition with online motion measurements or CBCT acquisition during arc treatment delivery. The online motion measurement typically requires fiducial markers and the invasive operation may not be always desirable, and therefore intrafraction CBCT imaging [7–10] may be more appropriate to obtain intrafractional localization errors. Meanwhile, for prostate registration between planning CT and CBCT images, statistically insignificant variations were reported between gray value correlation and automated bone-anatomy matching followed by therapist's manual adjustments [11], indicating the validity of the gray value correlation technique.

It would be valuable to retrospectively verify the target registration accuracy by comparing planning CT and intreatment CBCT images acquired during volumetric modulated arc therapy (VMAT). To the authors' knowledge, the calculation of time-averaged intrafractional tumor localization errors based on the CBCT imaging during VMAT has not been reported. The purpose of this study was to provide a first result of the intrafractional prostate localization errors and desirable PTV margins by comparing the prostate images of the planning CT and the CBCT during VMAT delivery using three-dimensional image cross-correlation.

## 2. Methods and Materials

### 2.1. Patients

Fourteen prostate cancer patients were treated with VMAT from May to December 2010. Patient characteristics are as follows: age at diagnosis (median): 73 years (range: 59–82); initial prostate-specific antigen (median): 8.25 ng/mL (range: 4.32–47.56); clinical T stage: T1c in 5, T2a in 2, T2b in 1, T2c in 2, and T3 in 4 patients; the Gleason score (sum): 6 in 2, 7 in 7, and 8 in 5 patients. Eleven patients had neoadjuvant hormonal therapies. Twelve plans were created by Pinnacle v9.0 (Philips, Eindhoven, Netherland), while two plans were created by Monaco 3.1 (Elekta AB, Stockholm, Sweden). For both, a single arc treatment from −179 to +179 degrees (clockwise) was employed with 76 Gy in 38 fractions to PTV (D95% prescription). Every patient was treated in supine position with a foot stand for intrafractional fixation. Oral intake of mosapride citrate hydrate after each meal was recommended for regular bowel movements. Written informed consent was obtained from each patient prior to the treatment.

### 2.2. IGRT Procedure for Treatments

Immediately before treatment, CBCT images were acquired by X-ray volume imaging (XVI) v4.2 equipped with Elekta Synergy linear accelerator and a standard patient couch (Elekta AB, Stockholm, Sweden). The registration was performed between the planning CT with a slice thickness of 2 mm and the pretreatment CBCT images with a cubic voxel size of 0.52 mm. The chamfer matching (bone matching) was employed first, and, then, a slight manual correction was made if the prostate image matching was incomplete by bone matching only. Thereafter, the patient couch was adjusted according to the registration result. Rotational angle correction was disregarded in this study because our standard couch supported translation along each axis only.

### 2.3. Data Acquisition during Treatment

The XVI system did not allow intrafraction CBCT imaging, and, therefore, we employed cine-mode projection imaging during VMAT delivery in order to perform CBCT imaging, where the XVI flat panel imager operated at a resolution of 512 × 512 with a pixel size of 0.52 mm at the isocenter at a fixed frame rate of 5.5 fps. The intrafraction CBCT with a cubic voxel size of 1 mm was reconstructed by an inhouse program based on the algorithm developed by Feldkamp et al. [15] and by Webb [16].

### 2.4. Evaluation

The planning CT data were isotropically resampled by 1 mm pitch to equalize the voxel dimensions with the CBCT data. A three-dimensional cross-correlation between the prostate images of the resampled planning CT and the intrafraction CBCT was calculated in each fraction. The translational positioning errors were calculated by searching the maximum of the cross-correlation indices by a resolution of 1 mm in the three-dimensional space where the prostate volume is located. Parabolic interpolation was further applied to search the maximum correlation, thereby providing higher resolution of the calculated positioning errors. Rotational setup errors were not reported in this study because our couch provides translational movement only.

Subsequently, PTV margin was obtained in three orthogonal directions by the van Herk formula, 2.5Σ + 0.7σ [12, 13], and its variant, 2.1Σ + 0.7σ [14], for 90% of patient population receiving at least 95% of the prescribed dose, where Σ stands for the standard deviation of mean localization errors among fractions for each patient and σ is the root mean square of the standard deviation of the localization errors among fractions for each patient. The original van Herk formula employed a spherically symmetric model and the variant used an anisotropic Cartesian coordinate model.

## 3. Results

**Figure 1:**
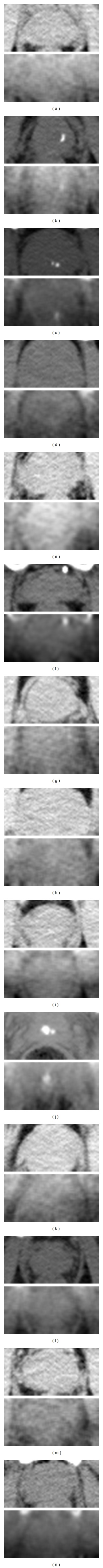
Prostate axial images of the planning CT and intrafraction cone-beam CT (CBCT) during volumetric modulated arc therapy for 14 prostate patients. The planning CT data were isotropically resampled by 1 mm pitch to equalize the voxel dimensions with the CBCT data. Three-dimensional image cross-correlation of the prostate volume between the planning CT and the CBCT data was calculated in each fraction and the translational positioning errors were calculated by searching the maximum of the cross-correlation.

**Figure 2:**
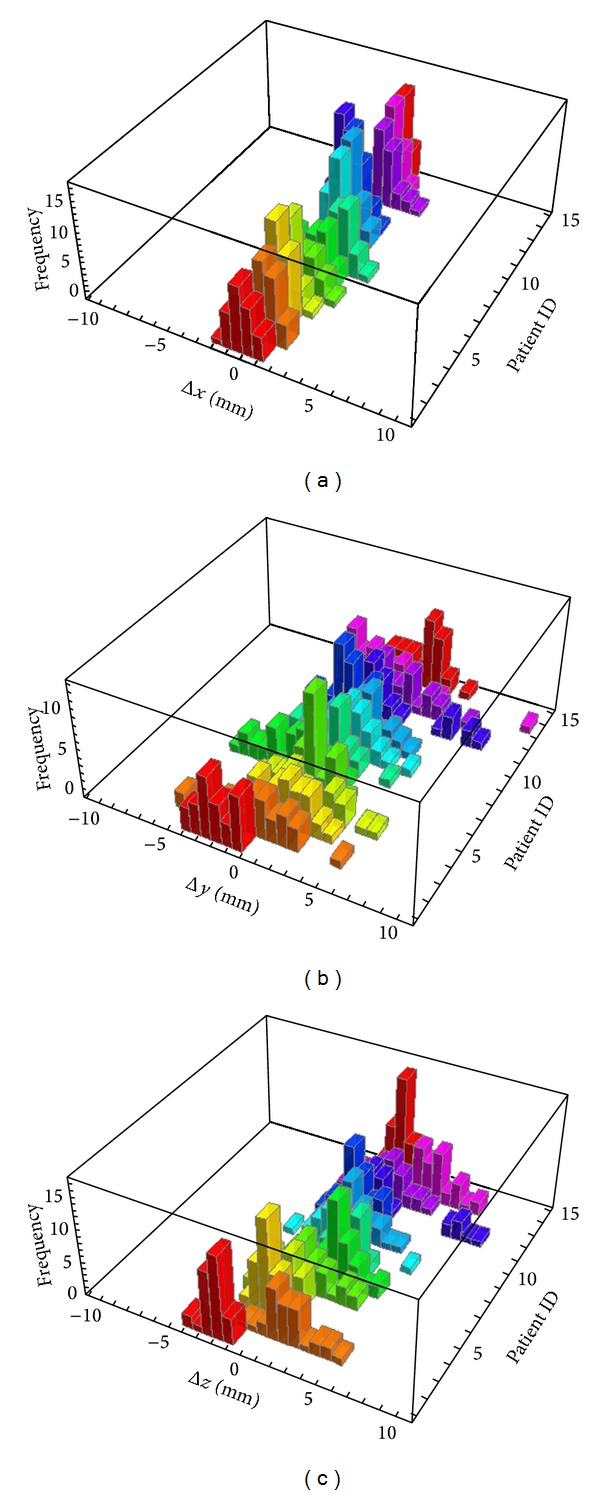
Histograms of calculated localization errors relative to the planning CT isocenter in (a) *x*, (b) *y*, and (c) *z* directions for 14 prostate cancer patients, with *x*-axis going from left to right, *y*-axis going from anterior to posterior, and *z*-axis going from cranial to caudal directions.

The beam delivery time ranged from 120.5 to 197.0 seconds with a median of 133.3, whereas monitor units varied from 462.1 to 742.6 with a median of 602.7. Ten minutes were always allocated for each patient from entering the linac room to leaving the room.  Figure 1 shows planning CT and intrafraction CBCT axial images during VMAT delivery for 14 prostate cancer patients. Figures 2(a)–2(c) show histograms of calculated localization errors in *x*, *y*, and *z* directions for each fraction of 14 prostate cancer patients, with *x*-axis going from left to right, *y*-axis going from anterior to posterior, and *z*-axis going from cranial to caudal directions.

Table 1 shows resulting intrafractional prostate localization errors and PTV margins calculated by the van Herk formula and its variant, again with *x*-axis going from left to right, *y*-axis going from anterior to posterior, and *z*-axis going from cranial to caudal directions. Mean shows patient average of mean localization errors among fractions for each patient. The definitions of Σ and *σ* were described earlier. Calculated PTV margins were approximately 2 mm in lateral direction and 4 mm in craniocaudal and anteroposterior directions.

## 4. Discussion

The calculated mean error in each direction was less than 1 mm as shown in Table 1. The present result may include mechanical errors caused by the treatment couch. Considering that the couch drive mechanism has a translation resolution of 1 mm, it may be concluded that the system worked properly within its precision. Calculated Σ and *σ* correspond to systematic and random errors, respectively [12, 13], and we obtained relatively larger errors in anteroposterior and craniocaudal directions compared to errors in lateral direction, which agrees with previous reports [1, 3, 5]. Because of the spatial relationship between prostate and rectum on a sagittal plane, direct mechanical force may lead to prostate movement toward anteroposterior and craniocaudal directions, which may explain the above results.

We have employed two different formulas for calculating the PTV margin, where the original van Herk formula employed a spherically symmetric model and the variant used an anisotropic Cartesian coordinate model which may provide more accuracy. Unless Σ exceeds 2.5 mm (which is unlikely under prostate CBCT image guidance), the difference between the two formulas is less than 1 mm and is therefore considered negligible. In our facility, an isotropic PTV margin of 5 mm was employed except in a posterior margin of 4 mm, which is justified by the calculated results shown in Table 1. In other words, our prostate registration using bone matching followed by slight manual correction is practically self-consistent with the current margin prescription employed in our facility. Meanwhile, satisfying biochemical control with few serious adverse events has been observed thus far.

It was reported that prostate displacements of greater than 3 mm were detected at 5 min after initial alignment in 13% of all the fractionated deliveries and increased to 25% by 10 min [5]. We can therefore anticipate that minimizing the treatment time may also minimize intrafraction registration errors. Besides, it is known that VMAT provides less treatment time compared to other techniques. For example, in this study, the VMAT treatment time ranged from 90 to 130 seconds, which is much faster than the five-field conformal radiotherapy mentioned above. Consequently, VMAT may be the most appropriate delivery option for minimizing intrafraction registration errors.

Limitations of the present procedure may be that the intrafraction CBCT imaging does not provide real-time tumor position but only time-averaged position data and a small dose (typically an order of 1 cGy) will be absorbed in a patient body. Nevertheless, the present analysis may be useful to determine a reasonable margin in an institution. It is also important to note that no delineation uncertainty was considered in the margin calculations. This may lead to an underestimation of the PTV margin [17].

In conclusion, time-averaged localization errors were calculated using cross-correlation of the prostate organ images between planning CT and intrafraction CBCT, and PTV margins were derived using the van Herk formula as well as its variant in three orthogonal directions. It was confirmed that our margin prescription is self-consistent with our prostate registration procedure. Lastly, the proposed procedure is fully noninvasive thereby providing much wider applicability.

## Figures and Tables

**Table 1:** Localization errors and planning target volume (PTV) margin calculated by the van Herk formula and Yoda's variant using 14 prostate patient data, with *x*-axis going from left to right, *y*-axis going from anterior to posterior, and *z*-axis going from cranial to caudal directions. Mean shows patient average of mean localization errors among fractions for each patient. Σ shows the standard deviation of mean localization errors among fractions for each patient. *σ* shows the root mean square of the standard deviation of the localization errors among fractions for each patient.

	*x* (mm)	*y* (mm)	*z* (mm)
Mean	0.28	0.49	0.79
Σ	0.67	1.22	1.38
*σ*	0.66	1.85	1.35
Van Herk [12, 13]	2.1	4.3	4.4
Yoda and Nakagawa [14]	1.9	3.9	3.9
